# Longitudinal study of *Plasmodium falciparum *and *Plasmodium vivax *in a Karen population in Thailand

**DOI:** 10.1186/1475-2875-7-99

**Published:** 2008-06-02

**Authors:** Waraphon Phimpraphi, Richard E Paul, Surapon Yimsamran, Supalarp Puangsa-art, Nipon Thanyavanich, Wanchai Maneeboonyang, Sutthiporn Prommongkol, Samarn Sornklom, Wutthichai Chaimungkun, Irwin F Chavez, Herve Blanc, Sornchai Looareesuwan, Anavaj Sakuntabhai, Pratap Singhasivanon

**Affiliations:** 1 Department of Tropical Hygiene, Faculty of Tropical Medicine, Mahidol University, Bangkok, Thailand; 2 Institut Pasteur, Laboratoire de Génétique de la réponse aux infections chez l'homme, 28 rue de Dr. Roux, F-75724, Paris cedex 15, France; 3 Department of Clinical Tropical Medicine, Faculty of Tropical Medicine, Mahidol University, Bangkok, Thailand

## Abstract

**Background:**

Clinical case treatment of malaria infections where *Plasmodium falciparum *and *Plasmodium vivax *are sympatric has achieved effective reductions in *P. falciparum *prevalence and incidence rates, but has been less successful for *P. vivax*. The high transmissibility of *P. vivax *and its capacity to relapse have been suggested to make it a harder parasite species to control.

**Methods:**

A clinical malaria case treatment programme was carried out over a decade in a Karen community composed of seven hamlets on the Thai-Myanmar border.

**Results:**

From 1994 to 2004, prevalence rates of both *P. falciparum *and *P. vivax *decreased by 70–90% in six of the seven study hamlets, but were unchanged in one hamlet. Overall, incidence rates decreased by 72% and 76% for *P. falciparum *and *P. vivax *respectively over the period 1999–2004. The age-incidence and prevalence curves suggested that *P. vivax *was more transmissible than *P. falciparum *despite a greater overall burden of infection with *P. falciparum*. Male gender was associated with increased risk of clinical presentation with either parasite species. Children (< 15 years old) had an increased risk of presenting with *P. vivax *but not *P. falciparum*.

**Conclusion:**

There was a considerable reduction in incidence rates of both *P. vivax *and *P. falciparum *over a decade following implementation of a case treatment programme. The concern that intervention methods would inadvertently favour one species over another, or even lead to an increase in one parasite species, does not appear to be fulfilled in this case.

## Background

Over the last decade in Thailand, a concerted programme at the national level of clinical case treatment coupled with vector control programmes has led to a greatly reduced burden of malaria [1], restricting malaria transmission to the border provinces. The Tak Malaria Initiative (TMI) sought to address the malaria problem in one of these border provinces, Tak, implementing a system of early diagnosis and treatment with mefloquine-artesunate combination therapy [2]. This initiative proved most successful in reducing morbidity and mortality of *P. falciparum *but had seemingly little impact on *P. vivax*. *Plasmodium vivax *accounts for over half of all malaria infections outside Africa and in 2002 the Multilateral Initiative on Malaria convened a special conference, "Vivax Malaria Research: 2002 and Beyond" [3]. The conference highlighted the disparity of knowledge and research effort concerning these two major malaria parasite species and called for increased effort to readdress this situation, including improving comprehension on the epidemiology of *P. vivax*.

Outside Africa, *P. falciparum *often coexists with *P. vivax*, and as noted above in the TMI, there have been many cases where *P. vivax *prevalence has remained unchanged, or even increased, despite a drop in *P. falciparum *[4,5]. This outcome goes against general predictions that current principle intervention strategies would have a greater impact on *P. vivax *than *P. falciparum *[6]. The reasons for such species-specific patterns are unclear and both mosquito and drug treatment explanations have been invoked [7,8]. It has been suggested that the biology of *P. vivax *makes it a harder species to control and one that will require a different strategy to that of *P. falciparum *[7]. The existence of exo-erythrocytic hypnozoite stages enables *P. vivax *to avoid drugs targeting blood stage parasites. *Plasmodium vivax *gametocytes are produced at very early stages of blood infection, even directly from hypnozoites and thus may enable transmission to mosquitoes before any drug treatment is delivered. The sporogonic development of *P. vivax *within the mosquito is the fastest of all malaria spp. infecting humans [9], taking on average 11 days to complete. Such differences may indeed enable *P. vivax *to be less affected by an intervention strategy targeting *P. falciparum*, but do not explain its observed increases in prevalence in areas sympatric for *P. falciparum *and *P. vivax*.

Increased prevalence of *P. vivax *has been noted especially in Thailand, increasing from 20% to 50% of cases over the last forty years [7]. Two major lines of argument have been used to explain this. Firstly it has long been recognized that there is competitive interaction between the two species within the human host during a co-infection [10-12]. It has more recently been suggested that the two species may negatively impact upon one another through the development of cross-species immunity [6] and there is evidence for species-transcending parasite density-dependent immune responses [13,14]. Thus, specifically targeting one species may release the other from competitive suppression. Indeed, emergence of a second species following drug clearance of the apparent species infection is well known [15,16]. Secondly, mosquito vector species may differ in their vectorial competence and capacity for *P. falciparum *and *P. vivax *[4,17,18]. Environmental changes that alter vector species composition could have a profound impact on parasite species abundance.

The relative contribution of these factors to the changing pattern of species abundance is difficult to ascertain and likely to be highly dependent on study site. However, it is clear that in order to develop appropriate intervention strategies to target both *P. falciparum *and *P. vivax *requires improved knowledge on basic *P. vivax *epidemiology. If, as has been suggested, the two parasite species impact upon each other when in co-existence, epidemiological studies need to consider both species simultaneously. This study reports on the basic epidemiological patterns of *P. falciparum *and *P. vivax *in a Karen community in Thailand on the border with Myanmar followed for a decade and receiving case treatment.

## Methods

### Study population and site

**Figure 1:**
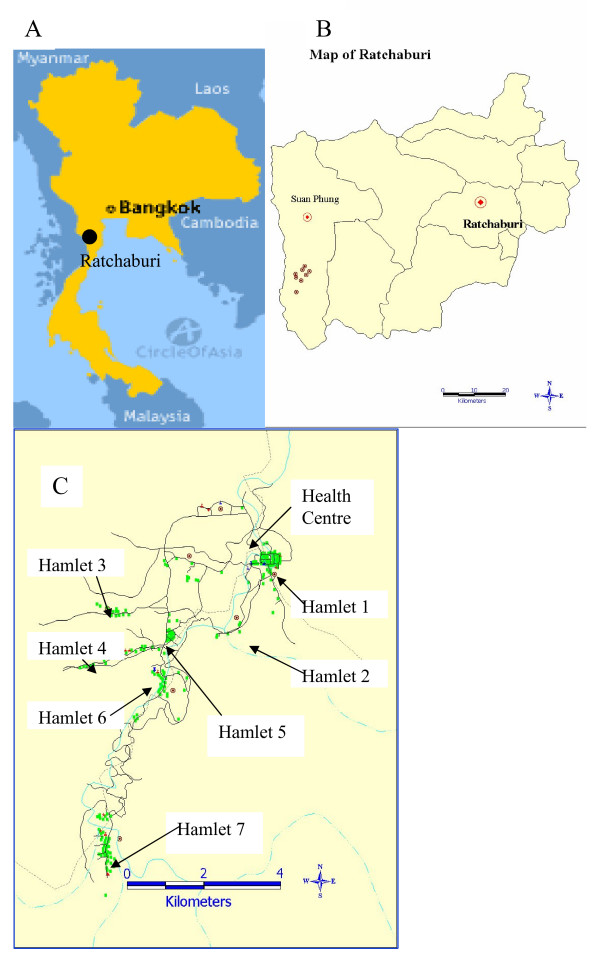
**Geographical localisation of study site.** (A) Ratchaburi in Thailand. (B) Suan Phung in Ratchaburi province and (C) Hamlets and health centre in study.

The study was conducted in a mountainous area of Suan Phung district, Ratchaburi province, Thailand (Figure 1). Suan Phung is a small district situated near the Thai-Myanmar border surrounded by the long Tanaosri ranges on the western side. The Tanaosri subdistrict is located at the southern part of Suan Phung, approximately 163 km west of Bangkok. Suan Phung currently has a population of 5,368, has been growing in population number over the last decade and is made up of a group of four closely related ethnic groups, the majority of which are Karen (85%), some Thai (14%) and the rest Mon and Burmese (1%). The majority of the both male and female labour forces are hunters-gatherers with limited agricultural practice. A census of the population was carried out in 1994/5, 1998, 2001 and 2004. Overall, a total of 3,484 villagers of all ages (Table 1) living in seven hamlets were recruited for the study (Figure 1). An initial survey during the 1994/5 census recruited 1,104 individuals and this number was increased to 1,469 by the 1998 census, 3,079 by the 2001 census and 3,484 by the 2004 census. Individuals wishing to participate in the study were recruited at each census and between times by presentation at the clinic. The project protocol and objectives were explained to the population and informed consent was individually obtained from all study participants or their parents. Ethical permission for the study was granted by the Ethical Committee of the Ministry of Public Health of Thailand. In this study site, the only two mosquito vector species are *Anopheles minimus *and *Anopheles maculatus*, which are present throughout the year and are predominantly exophagic [18]. The rainy season occurs from June to September. Although bed nets have been promoted, their use remains limited; reliable information on their regular use was obtained from only 726 individuals.

**Table 1:** Age and sex distribution of the population studied at the time of the 2004 census.

Age	N	male	female
< 1	33	19	14
1–4	462	241	221
5–9	510	262	248
10–14	455	224	231
15–24	730	357	373
25–39	740	362	378
40–59	428	218	210
≥ 60	126	54	72
Total	3484	1737	1747

### Data collection

Both active and passive case detection methods were used to collect the data. Two cross-sectional serial surveys were carried out, in 1994–5 and in 2003–4 and the incidence of malaria was measured from June 1998 to February 2005 in a health centre constructed for the site.

#### Active case detection

In order to determine the prevalence of malaria, a malaria survey (thick and thin smears) was carried out every month from June 1994 to May 1995, covering a population of 1,104 individuals in the seven hamlets. A second cross-sectional survey was carried out every 45 days from June 2003 to May 2004 in all hamlets, covering 2,663 subjects who agreed to participate.

#### Passive case detection

Clinical malaria episodes were defined as the presence fever (axillary temperature > 37.5°C) or fever-related symptoms including headache, back pain, chills, myalgia, nausea, and vomiting associated with a slide positive for blood-stage asexual or sexual *P. falciparum*, *P. vivax, Plasmodium malariae *and *Plasmodium ovale *parasites at any density. To determine the correct number of independent clinical episodes, individuals presenting on consecutive occasions with blood-stage malaria parasite of the same species within 30 days (and also 90 days for *P. vivax*) after treatment of the initial infection were excluded from the analysis. The 30- and 90-day criteria were set to make sure that the most recent episode of malaria is a new one and not from a previous infection. Relapse rate of vivax in Thailand is quite high (even if drug compliance and treatment completion is high) and it is thus questionable to assume that an episode 30 days after a previous vivax infection is a new one; hence we additionally analyse using a 90-day exclusion time. On the other hand, since the drug regimen for falciparum malaria is more efficacious, 30 days is sufficient to rule out recrudescence. The "unexcluded" incidence rates were additionally provided. Individuals presenting on consecutive occasions with non-malaria fever (or aforementioned symptoms) within seven days following first presentation were likewise excluded from analyses. In addition, initial parasite negative visits were excluded if followed by a parasite-positive visit within the following two days. All positive malaria cases were treated with appropriate antimalarial treatment according to the recommendation of the Malaria Division, Ministry of Public Health. Subjects diagnosed with having *P. falciparum *were treated with a single dose of 250 mg mefloquine tablets and primaquine depending on age (Table 2a) and, when diagnosed positive for *P. vivax*, treated with chloroquine (250 mg) and primaquine for four days following the schedule in Table 2b. Self-treatment is considered to be rare in the study area, because the only other access to treatment is a government clinic with which the study has good communication concerning malaria treatment of the study site population. Early treatment failure (ETF) and Late treatment failure (LTF) were defined as presentation with the same malaria species within seven days and seven to 30 days post-treatment, respectively.

**Table 2:** Drug treatment schedules and doses

(a) *P. falciparum*										
Patient group	Mefloquine (250 mg tablet)	Primaquine (mg)								

< 1 year	1/2	-								
1–3 years	3/4	5								
4–8 years	1 1/2	10								
9–14 years	2	15								
15+ years	3	30								

(b) *P. vivax*										

Patient group	Day 1				Day 2		Day 3		Day 4	

	C*	C*	C*	P^+^	C*	P^+^	C*	P^+^	C*	P^+^
< 6 m	1/2	-	-	-	1/2	-	-	-	-	-
6–11 m	1	-	-	-	1/2	1	1/2	-	-	-
1–3 y	1	1	-	2.5	1	2.5	1	2.5	1	2.5
4–8 y	1	1	1	5	1	5	1	5	1	5
9–14 y	2	2	-	10	1	10	1	10	1	10
15+y	2	2	2	15	2	15	2	15	2	15

m – months; y – years; C – chloroquine phosphate (250 mg); P – primaquine; * - tablet; ^+ ^- mg

In all cases parasite positivity was established as follows. Thick and thin blood films were prepared and stained by 3% Giemsa. Blood films were examined under an oil immersion objective at × 1,000 magnification by the trained laboratory technicians and 200 thick film fields were examined before films were declared negative. Parasite species were identified on thin films and densities (per μl) were calculated from thick film by establishing the ratio of parasites to white blood cells (WBC) after at least 200–500 WBCs had been counted and then multiplying the parasite count by 8,000, the average WBC count per μl of blood. Asexual and gametocyte stages were recorded separately.

### Data and statistical analyses

Statistical analyses and model fitting were conducted using the statistical package Genstat 7.1.

#### Cross-sectional study analyses (active case detection in 1994 and 2004)

Factors affecting prevalence rates of both *P. falciparum *and *P. vivax *and changes over time were analysed by fitting a GLMM (Generalised Linear Mixed Model) with binomial error structure. Individual person was fitted as a factor in the random model to take into account repeated samples from the same individual. The cross-sectional surveys (1994 and 2004) were first analysed separately and then together. Hamlet (7), gender (male/female) and age (< or ≥ 15 years old) were fitted as explanatory variables and model fitting proceeded by progressively removing non-significant factors from the full model that included all possible variable interaction terms. Inclusion of such interaction terms was considered important as it has been reported on numerous occasions that gender and age-specific behaviour can influence exposure and this can vary locally depending on the nature of the environment. Age was additionally fitted as a factor with eight groups (< 1, 1–4, 5–9, 10–14, 15–24, 25–39, 40–59 and 60+ years of age), and as a continuous variable, but both proved less explanatory (lower adjusted r^2^) than age defined as < or ≥ 15 years old. Because the data were over-dispersed a dispersion parameter was estimated. Wald statistics, which approximate to a χ^2 ^distribution, were established. Odds ratios with 95% confidence intervals (CI_95%_) are given.

#### Clinical case analyses (passive case detection)

Factors influencing incidence rates and parasite density of either *P. falciparum *or *P. vivax *were analysed by fitting a GLMM with a Poisson error structure. Individual person was fitted as a factor in the random model to take into account repeated presentations from the same individual. Factors include year (1999–2004), month (12), hamlet (7), and gender, with age factored into two groups (< 15 and ≥ 15 years of age). Initially a full model including age-gender-hamlet interactions was fitted and then the model was refined to include only statistically significant parameters and interactions. The seasonality of transmission in this region varies annually and thus month was considered only via its interaction with year. Because the data were over-dispersed a dispersion parameter was estimated. Wald statistics, which approximate to a χ^2 ^distribution, were established. The effect of bed net use was analysed using the reduced data set (N = 726), incorporating bed net use as an extra explanatory factor and analysed as above. Relative Risk with CI_95% _is given.

Spearman's Rank correlation was used to establish the correlation between the monthly numbers of cases of *P. vivax *(or *P. falciparum*) that occurred within 90 days following treatment of *P. falciparum *(or *P. vivax*) with the numbers of cases of *P. vivax *(or *P. falciparum*) occurring independently of treatment.

#### Attributable fraction

A clinical case of malaria is often diagnosed if the individual presents with fever, headache or other symptoms associated with malaria (such as chills, pains etc) and has a positive blood slide. The fraction of fevers (or other malaria-associated symptoms) attributable to malaria can be simply calculated as $AF=\frac{P_{f}-P_{a}}{1-P_{a}}$ where *P*_*f *_is the proportion of fevers with parasites present and *P*_*a *_the proportion of asymptomatic but parasite positive individuals, sampled from the general population. However, in areas endemic for malaria, the proportion *P*_*a *_may be very high and thus an alternative formula is widely used where $AF=1-\frac{1}{OddsRatioP_{f}:P_{a}}\times P_{f}$ where *Odds Ratio P*_*f*_:*P*_*a *_is the proportion of odds of having parasites with fever over having parasites without fever [19]. Here using the cross-sectional survey performed in 2003–2004, with clinical case data from the corresponding period, the proportion of clinical presentations with malaria was compared with the calculated attributable fraction.

## Results

### Active case detection: changes in gross prevalence rates over the decade of intervention

**Figure 2:**
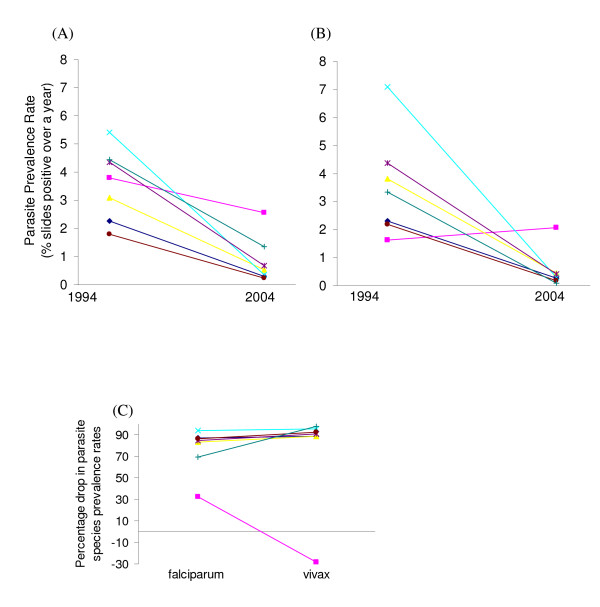
**Schematic diagram of (A) *P. falciparum *and (B) *P. vivax *gross prevalence rates and (C) Percentage drop in gross prevalence rates in seven hamlets in Suan Phung following a decade of clinical case treatment.** Connecting lines are illustrative rather than indicative of any linear relationship. Hamlet code: 1: Blue diamond ◇, 2: Pink square □, 3: Yellow triangle △, 4: Light blue cross ×, 5: Purple star *, 6: Brown circle ●, 7: Green plus +.

**Figure 3:**
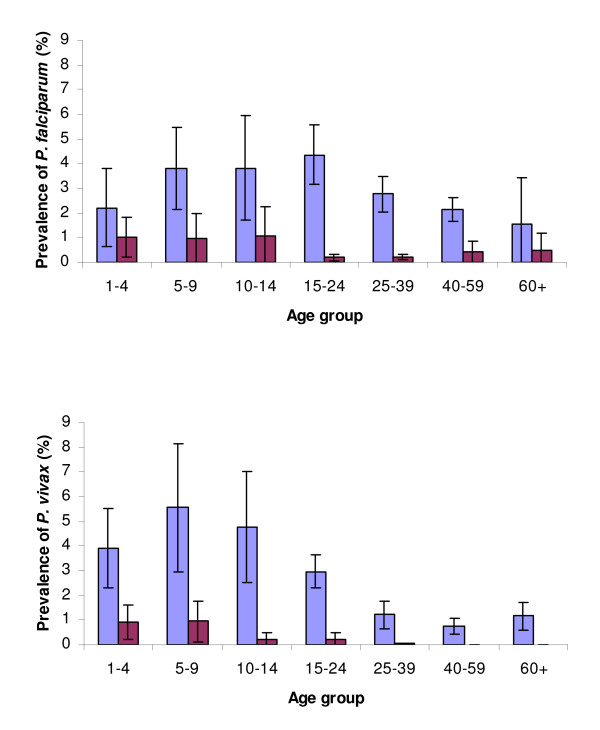
**Age-specific prevalence rates of *P. falciparum *and *P. vivax *in 1994 (light blue) and 2004 (purple).** Shown are the weighted mean (± 95% Confidence intervals) percentage of each age group infected during cross-sectional surveys in 1994 and 2004 across the seven study hamlets.

In the cross-sectional study in 1994, a total of 9,417 slides were read from 1,104 individuals for whom complete information on age, gender and hamlet were available. *Plasmodium falciparum *prevalence rates varied seasonally from 0.5% in February to 7% in June, varied among hamlets (P < 0.001) (Figure 2a) and were higher in males than females (3.41% vs. 1.95%) (OR = 1.90 [CI_95% _1.43–2.37] P = 0.003). However, there was a significant gender-age group interaction whereby adult males (≥ 15 years old) had higher prevalence rates than male children (< 15 years old), whereas adult females had lower prevalence rates than female children (P < 0.001). Analysing by gender revealed that although female children had increased odds of being infected with *P. falciparum *(OR = 1.50 [CI_95% _1.09–2.41] P = 0.035), male children did not in fact have significantly lower odds of infection than adult males (OR = 0.71 [CI_95% _0.54–1.04] P = 0.093). For *P. vivax*, by contrast, prevalence rates were significantly higher in the children irrespective of gender (OR = 2.89 [CI_95% _2.30–3.89] P < 0.001); gender was however important *per se *with males having a slightly higher prevalence rate than females (1.75% vs. 1.06%; OR = 1.74 [CI_95% _1.31–2.16] P < 0.001). *P. vivax *prevalence rates again differed significantly among hamlets (P < 0.001) (Figure 2b). Seasonal variation in prevalence rates was less marked for *P. vivax*, varying from 1.4% in February to 4.5% in May. Age-prevalence profiles differed between the parasite species, where peak prevalence of *P. vivax *occurred at 5–9 years old with a rapid decline after 14 years of age, whereas *P. falciparum *prevalence rates increased steadily to a peak at 15–24 years old (Figure 3). There were 31 *P. malariae *and one *P. ovale *positive slides. There were 16 mixed infections of *P. falciparum-P. vivax*, 2 mixed infections of *P. falciparum-P. malariae *and 1 of *P. malariae-P. vivax*.

In 2004, a total of 13,721 slides were read from 2,663 individuals. *Plasmodium falciparum *prevalence rates still varied among hamlets (P < 0.001) (Figure 2a) and were higher in males *vs*. females (0.98% *vs*. 0.33%) (OR = 2.58 [CI_95% _2.26–2.90] P < 0.001) and children *vs*. adults (0.97% vs. 0.70%) (OR = 2.86 [CI_95% _2.52–3.29] P < 0.001). There was, however, no longer any gender-age interaction. For *P. vivax*, there remained significant variability among hamlets (P = 0.003), largely due to hamlet 2 (Figure 2b) and significantly higher rates in children than adults (0.70% vs. 0.07%) (OR = 8.71 [CI_95% _7.30–10.79] P < 0.001). Males and females, however, did not differ (0.5% *vs*. 0.29%)(P = 0.14). Seasonal variation was likewise less marked, varying from 0.1% in April to 1.5% in June for *P. falciparum *and from 0.2% to 0.6% for *P. vivax*. Age-prevalence profiles similarly levelled off across age groups, although *P. vivax *prevalence remained concentrated in the < 15 year olds (Figure 3). There were no positive slides for *P. malariae *or *P. ovale *and no mixed infections.

As evident from Figure 2c, gross prevalence rates of both *P. falciparum *and *P. vivax *decreased over the decade by 70–98% (P < 0.001 for both species) in six of the seven hamlets. Hamlet 2, however, showed only a small decrease in *P. falciparum *prevalence rates (32%) and a 28% increase in *P. vivax *prevalence rates. These changes in hamlet 2 were not significant (*P. falciparum *P = 0.59; *P. vivax *P = 0.69); notably only 20 individuals in hamlet 2 participated in the 1994 study compared with 209 in the 2004 study.

### Passive case detection: incidence rates

From 1999 to 2004 (six years) there were 17,454 independent clinical presentations by 2,515 individuals (Range 1–47 per person; mean 6.9 and median 5). Of these 2,136 were positive for *P. falciparum*, presented by 1,048 individuals (Minimum of 1 and a maximum of 13 positive presentations per person; mean 2.0 and median 1). Fever (94% of all malaria parasite positive infections) and headache without fever (3.3%) were associated with the majority of infections with *P. falciparum*. There were 1,142 cases of *P. vivax *(948 cases using the > 90 day novel infection criterion), presented by 582 individuals (Minimum of 1 and a maximum of 12 positive presentations per person; mean 2.0 and median 1). In addition there were 28 cases of *P. malariae *and five cases of *P. ovale*. Fever (95.3% of all positive infections) and headache without fever (2.1%) were associated with the majority of infections with *P. vivax*. A total of 3,246 infections by any malaria parasite spp. were presented by 1,298 individuals at any time over the period of study. Some 332 individuals had independent infections of both *P. falciparum *and *P. vivax*, 250 were only ever infected with *P. vivax *and 716 only ever with *P. falciparum*. Only 32 *P. falciparum – P. vivax *and three *P. falciparum – P. malariae *mixed infections were observed. Over all hamlets incidence rates for clinical cases positive for *P. falciparum *decreased from 248 (CI_95% _122–374) per 1,000 person-years in 1999–2001 to 69 (CI_95% _47–92) in 2002–2004 and for *P. vivax *rates decreased from 139 (CI_95% _65–214) to 33 (CI_95% _19–46).

Exclusion of repeated presentation within 30 days led to the exclusion of 474 cases of *P. falciparum *and 18 cases of *P. vivax *(194 cases using the > 90 day novel infection criterion). There was a clear decrease in this number from year 2002 for *P. falciparum *(1999–2001 Mean 103 CI_95% _91–115 *vs*. 2002–2004 Mean 55 CI_95% _43–67). Incidence rates using the full "unexcluded" data set were 300 (CI_95% _148–452) per 1,000 person-years in 1999–2001 and 86 (CI_95% _59–113) in 2002–2004 for *P. falciparum *and for *P. vivax *the incidence rates were 142 (CI_95% _66–218) in 1999–2001 and 33 (CI_95% _20–47) in 2002–2004. There were eight cases of *P. falciparum *ETF and 78 cases of LTF. There were no cases of treatment failure for *P. vivax*.

**Figure 4:**
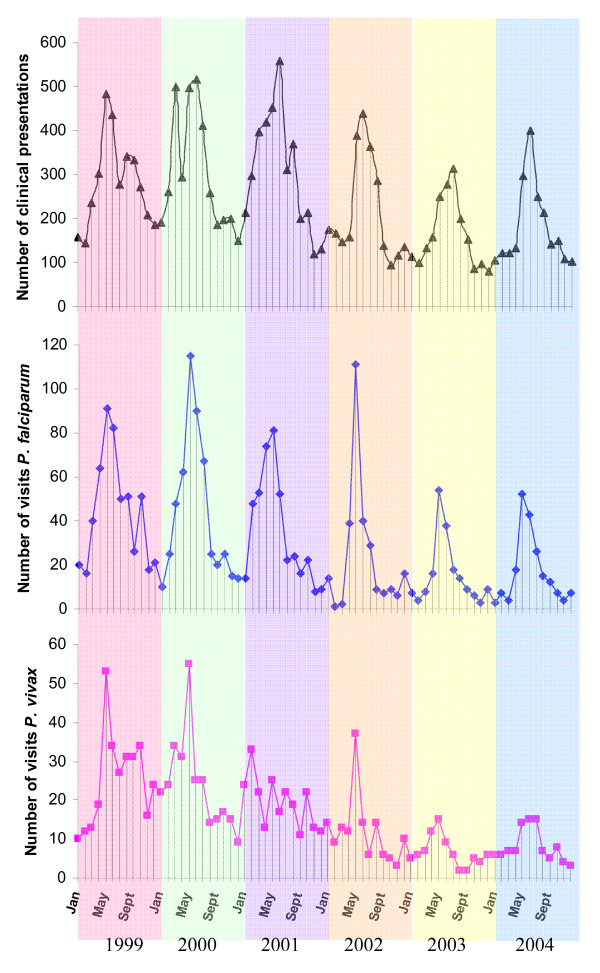
**Summary of the total number of monthly clinical presentations, those positive for *P. falciparum *and those positive for *P. vivax *from Jan 1999 to Dec 2004.** Background colour shading delimits year.

There was distinct seasonality in clinical cases with annual peaks from April to June (Figure 4). *Plasmodium falciparum *clinical cases peaked annually in May, accounting for 17–39% of the total number of annual cases. The annual peak of clinical *P. vivax *cases in May was apparent but less marked, but still accounted for 10–25% of annual cases. Examination of infections of one species following treatment of another, revealed only 19 cases of *P. vivax *within 30 days following treatment of *P. falciparum *and 16 cases of *P. falciparum *following treatment of *P. vivax*. Extending this period to 90 days revealed 87 cases of *P. vivax *occurring following treatment of a *P. falciparum *case and 47 cases of *P. falciparum *occurring after treatment of *P. vivax*. This number decreased from 37 and 55 in 1999–2000 to 4 and 6 in 2003–4 for *P. falciparum *and *P. vivax*, respectively. In an attempt to determine the likelihood that these "90-day post-treatment" cases arose from emerging co-infections rather than novel infections, their distribution over time was compared to that of novel "independent" cases by Spearman's Rank correlation. Although there was significant correlation between the distribution of post-treatment and novel "independent" cases (*P. falciparum *P < 0.001; *P. vivax *P < 0.001), correlation coefficients of *P. vivax *(post-treatment vs. "novel") and *P. falciparum *(post-treatment vs. "novel") were only 0.45 and 0.56 respectively. By comparison, the correlation coefficient between *P. vivax *and *P. falciparum *novel cases was 0.75.

**Figure 5:**
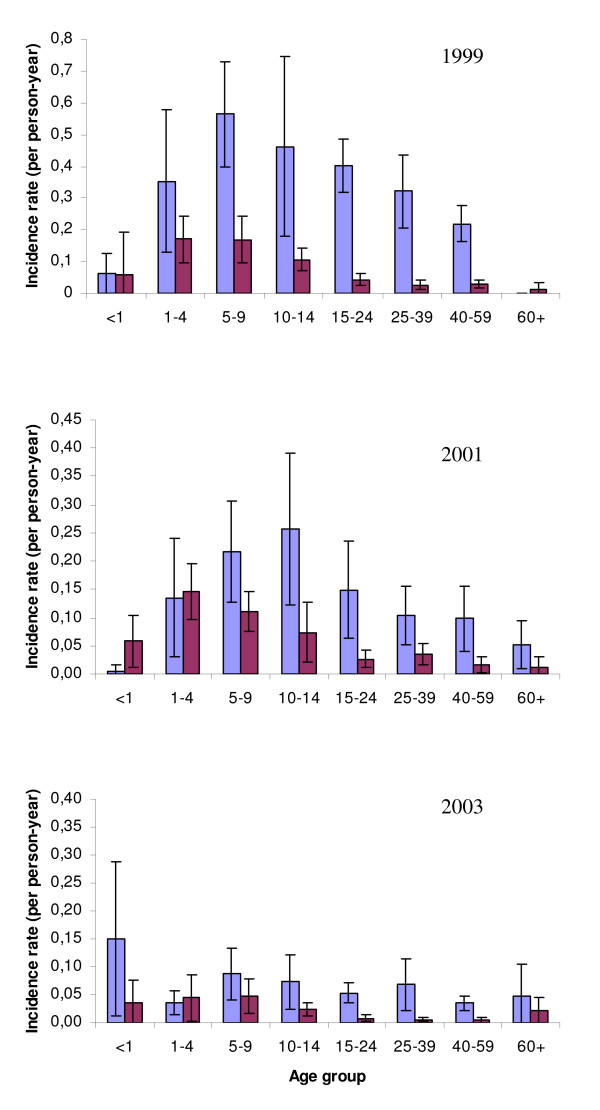
**Age-specific malaria incidence rates (light blue *P. falciparum*; purple *P. vivax*).** Shown are the weighted mean (± 95% Confidence intervals) number of clinical presentations per person-year in each age group infected across the seven hamlets in 1999, 2001 and 2003.

As with the cross-sectional studies, there was considerable variation in incidence rates among hamlets, being highest for both species in hamlet 2. Male gender was associated with increased risk of clinical presentation with either *P. falciparum *(RR = 1.42 [CI_95%_1.34–1.52] P < 0.001) or *P. vivax *(RR = 1.24 [CI_95%_1.16–1.33] P < 0.001). Children (aged < 15 years old) had an increased risk of presenting with *P. vivax *over adults (RR = 2.86 [CI_95% _2.63–3.12] P < 0.001), but not so for *P. falciparum *(P = 0.32). More detailed age-specific incidence rates showed that, as found in the cross-sectional surveys, peak incidence occurred in younger age groups (one to nine years old) for *P. vivax *than for *P. falciparum *(5–14 years old) (Figure 5). By 2003 age-specific differences in incidence rates became negligible for either species. Bed nets were not found to have significant impact on either *P. falciparum *(P = 0.059) or *P. vivax *(P = 0.86) incidence rates.

There was good concordance in the proportion of fevers that were found to be positive for malaria parasites and the fraction of fevers attributable to malaria as calculated using two methods that incorporate cross-sectional survey data (Table 3). This suggests that a large majority of infections lead to febrile episodes and that the proportion of infections that are asymptomatic is very small.

**Table 3:** Proportion of fevers positive for malaria compared with the fraction of fevers (and 95% confidence intervals) attributable to malaria in individuals less than and greater than 15 years old, calculated by Method 1: $AF=1-\frac{1}{OddsRatioP_{f}:P_{a}}\times P_{f}$ and Method 2: $AF=\frac{P_{f}-P_{a}}{1-P_{a}}$. *P*_*f *_is the proportion of fevers with parasites and *P*_*a *_the proportion of asymptomatic but parasite positive individuals.

Age (years)	Propn. fevers malaria positive	AF – Method1	AF – Method2
*Pfal*			
< 15	0.104	0.086 (0.072–0.103)	0.101 (0.098–0.103)
≥ 15	0.101	0.089 (0.072–0.11)	0.099 (0.097–0.100)

*Pviv*			
< 15	0.051	0.041 (0.031–0.053)	0.048 (0.045–0.050)
≥ 15	0.028	0.025 (0.016–0.038)	0.028 (0.027–0.028)

AF: Attributable Fraction. *Pfal*: *Plasmodium falciparum*; *Pviv*: *Plasmodium vivax*. See Methods for details.

### Parasite density and gametocyte parasite stages

Peak *P. falciparum *trophozoite densities were observed in one to nine year olds (55,409 ± 4,825 parasites/μl) and decreased steadily with age to level off in the > 25 year olds (12,402 ± 1,418 parasites/μl). *P. vivax *asexual parasite densities were lower than those of *P. falciparum *(8,105 ± 412 parasites/μl vs. 36,927 ± 2,419). Densities peaked in the 0–9 year olds (9,264 ± 548 parasites/μl) but decreased only slowly with age (> 25 years old 4116 ± 711 parasites/μl). For either species there were no differences among hamlets and densities oscillated over the years with an increase from 1999 to 2002 and then decreasing.

Of the 2,101 clinical presentations positive only for *P. falciparum*, 84 had gametocytes whereas of the 32 mixed *P. falciparum-P. vivax *infections only one had *P. falciparum *gametocytes and 10 had *P. vivax *gametocytes. Of the 1,110 *P. vivax *single infections 324 had gametocytes. There was thus no discernable effect of mixed infections on gametocyte production for either *P. falciparum *(P = 0.48) or *P. vivax *(P = 0.96). The proportion of *P. falciparum *and *P. vivax *infections with gametocytes was strongly affected by age: Children (< 15) had higher odds of having detectable gametocytes than adults (*P. falciparum *OR = 3.11 [CI_95% _2.37–4.52] P < 0.001; *P. vivax *OR = 1.56 [CI_95%_1.12–2.58] P = 0.02). The proportion of infections with gametocytes did not change over time. A mean of 35% (CI_95% _29–40%) of *P. vivax *infections had gametocytes over the six years and 6.4% (CI_95% _4.1–8.5%) of *P. falciparum *infections had gametocytes. *Plasmodium vivax *gametocyte densities were higher than those of *P. falciparum *(670/μl [CI_95% _550–790] *vs*. 332 [CI_95% _181–483]).

## Discussion

Over the decade from 1994–2004 there was a considerable reduction in both *P. falciparum *and *P. vivax *prevalence rates in this Karen community. In contrast to many reports on the consequences of clinical case treatment for relative parasite species composition [2,4,5], there were similar reductions in the prevalence rates of both *P. vivax *than *P. falciparum *(Figure 2c). Drug treatment in all cases followed the national guidelines and was specific to each parasite species. The absence of treatment failures for *P. vivax *suggests that drug resistance has not developed to chloroquine and primaquine, as has generally been found to be the case in Thailand over this period of study [20]. No changes in malaria mosquito vectors were observed in the study area, with *An. minimus *and *An. maculatus *remaining the only two vectors. Bed nets (unimpregnated) had, as elsewhere described in Thailand [21], little impact on incidence rates, most likely reflecting the exophilic behaviour of the mosquito [22].

This study provided an insight into key epidemiological parameters of both *P. falciparum *and *P. vivax *and how they changed with an overall decrease in malaria. In addition, several risk factors pertinent to the epidemiology of both *P. falciparum *and *P. vivax *were identified and notably these risk factors were not identical for both species. The majority of infections seemingly lead to a symptomatic episode, permitting comparison of data from the cross-sectional surveys and the clinical case detection. The clinical case study and the 1994 initial survey revealed that male gender was a risk factor for both *P. falciparum *and *P. vivax*. This risk was lost for *P. vivax *in 2004, most probably because of the greatly reduced burden of *P. vivax *infection and the relatively small risk previously incurred. Gender has been previously identified as a risk factor for *P. falciparum *infection in Thailand [21], where it was suggested to reflect an increased exposure to infection associated with adult male tendency to be active outdoors in the evening. Here the effect of gender was less important, which might reflect the absence of *Anopheles dirus*, a forest-dwelling mosquito that is a better vector than *An. minimus *or *An. maculatus*. Young age (here considered < 15 *vs*. ≥ 15 years old) incurred a considerable risk for *P. vivax *infection in both cross-sectional surveys and in clinical presentation. By contrast age did not incur a risk for *P. falciparum *infection except in the 2004 cross-sectional study.

Paradoxical parasite species-specific age-profiles for sympatric *P. falciparum *and *P. vivax *have been repeatedly noted [21,23,24], where peak prevalence and incidence of *P. vivax *occurs at an earlier age than for *P. falciparum *despite higher overall rates of *P. falciparum*. A lower average age of first infection would indicate that *P. vivax *is more transmissible and many features of its biology, most especially those involved in transmission to the vector (gametocyte productivity and duration of sporogonic development), would enable this. In addition, some evidence suggests that α^+^-thalassemia individuals are more susceptible to *P. vivax*, but protected from severe *P. falciparum *disease and that thus there may be some cross-species immunity [6,25]. Early age infection by *P. vivax *may thus reduce disease and infection rates due to *P. falciparum*. Here, peak rates of *P. vivax *occurred in the one to nine year olds until 2004, at which time rates were very low and imperceptibly different among the young age groups. *P. falciparum *prevalence rates decreased from the age of 25 year olds in 1994, then stabilized out at a peak in the 5–14 year olds until 2004 at which time rates became concentrated in the < 15 year olds. How to explain this apparent paradox of *P. vivax *imposing a lower burden of disease and yet predominately occurring in younger age groups than *P. falciparum *in a setting where there the proportion of asymptomatic infections for either species is very small. The proposed biological features making *P. vivax *more transmissible are evident here and α^+^-thalassemia occurs at a high frequency in this region. The proportion of infections with detectable gametocytes was five times higher for *P. vivax *than for *P. falciparum *and the gametocyte density considerably higher despite lower asexual densities. Moreover, that children had an increased risk of having infections with gametocytes for both species would be of greater significance for *P. vivax *where age was found to incur an increased risk of infection. Such transmission and infection advantages would be compatible with the observed younger infection age profile for *P. vivax*, and yet overall incidence rates are lower than for *P. falciparum*. This observed difference, however, may belie the actual relative abundance of the two species and a crucial contributing factor may be the suppressive effect of *P. falciparum *in a co-infection.

Several recent articles have provided an excellent review of the importance of mixed infections and their likely underestimation, especially in Thailand [26-28]. The capacity of one *Plasmodium *species to suppress another within the human is to some extent dependent on the infection status of each species, where both *P. falciparum *and *P. vivax *can dominate the infection. However, *P. falciparum *generally dominates when present [11,12]. Misdiagnosis of cryptic mixed infections as single species infections has obviously important consequences for establishing prevalence rates. Although PCR has to some extent improved capacity to detect cryptic infections, *P. vivax *may remain latent as hypnozoite stages in the liver until the *P. falciparum *infection has been cleared. Here, there were a large number of *P. vivax *cases that occurred following treatment of *P. falciparum*, as has been previously found in Thailand [15]. A lesser number of *P. falciparum *cases followed treatment of *P. vivax *infections. Unequivocally distinguishing novel from latent infections in this study site of seasonally variable but perennial transmission is not possible with the current study design. However, simple analysis showed that there was worse correlation between the monthly incidence of post-treatment and independent infections for each species than between independent infections of either species. Notably the correlation was poorest for *P. vivax *(independent vs. post-treatment). This would be consistent with at least a proportion of such post-treatment *P. vivax *infections being latent and not novel infections.

Patterns of reciprocal seasonality in parasite spp. rates have been interpreted as additional suggestive evidence for antagonistic interaction [24]. Such patterns are not, however, consistently found and will certainly depend on vector species activity. In this study peak incidence occurred in May for both species and accounted for a significant proportion of total annual disease incidence. Despite the overall low force of infection, such strong seasonality would increase considerably the possibility of concomitant inoculation of both species and thus the potential for antagonistic interaction. Both experimental infection and epidemiological studies suggest that *P. falciparum *has a greater suppressive potential [6,11,12,29] and the data in this study are consistent with this. Thus, the greater potential for transmission by *P. vivax *may in fact result in a greater number of infections, but which do not materialize as patent infections until later, if at all; it has been estimated that only 60% of *P. vivax *infections relapse in Thailand [16]. When delayed patency occurs, it is likely to be at a time less propitious for transmission. Such temporal heterogeneity in transmission intensity may be alleviated by non-overlapping spatial distribution of the parasite species, which can occur even at very local scales [30,31]. Only small differences in relative parasite species rates were, however, observed among the seven hamlets.

## Conclusion

In conclusion, in this study site where a programme of passive case detection and treatment of both *P. vivax *and *P. falciparum *was put into place, there has been a considerable reduction in incidence rates over a decade. The concern that intervention methods would inadvertently favour one species over another [6,7], or even lead to an increase in one parasite species, does not appear to be fulfilled in this case.

## Authors' contributions

WP and RP co-wrote the paper and analysed the data. SY, WC, IFC and HB managed the data set. NT, WM, SP, SS carried out the study and SL, AS and PS designed the study.
